# Pilot Study on the Effects of First-Line Antituberculosis Drugs and Their Combinations on Selected Reproductive Endpoints in Female Rats

**DOI:** 10.3390/life16060878

**Published:** 2026-05-24

**Authors:** Elif Esra Uyar, Bulent Yavuzer, Mansura Babayeva, Nurinisa Yucel, Murat Gunay, Halis Suleyman

**Affiliations:** 1 Department of In Vitro Fertilization, Clinic of Gynecology and Obstetrics, Lokman Hekim Istanbul Hospital, Istanbul 34912, Turkey; dresrauyar@gmail.com; 2 Department of Pharmacology, Faculty of Medicine, Erzincan Binali Yıldırım University, Erzincan 24100, Turkey; bulent.yavuzer@erzincan.edu.tr; 3 Department of Internal Medicine 2, Azerbaijan Medical University, Baku AZ1022, Azerbaijan; dr.babayevamansura@gmail.com; 4 Pharmacy Services Program, Vocational School of Health Services, Erzincan Binali Yıldırım University, Erzincan 24036, Turkey; nurinisa.yucel@erzincan.edu.tr; 5 Department of Medical Biochemistry, Faculty of Medicine, Erzincan Binali Yıldırım University, Erzincan 24100, Turkey; mgunay@erzincan.edu.tr

**Keywords:** anti-mullerian hormone, antituberculosis agents, ethambutol, female infertility, isoniazid, oxidative stress, prolactin, pyrazinamide, rifampicin, rats

## Abstract

Background: The reproductive toxicity of first-line antituberculosis drugs remains poorly understood, particularly when used in combination. Rifampicin, isoniazid, pyrazinamide, and ethambutol are essential in tuberculosis therapy, but their potential influence on female fertility is uncertain. This pilot study evaluated their effects, given alone or in dual, triple, and quadruple combinations, on oxidative stress, endocrine markers, and reproductive outcomes in healthy female rats. Materials and Methods: Ninety-six albino Wistar-type female rats were divided into sixteen groups of six animals each and treated with single, dual, triple, or quadruple regimens of first-line antituberculosis drugs for 28 days. After treatment, two sexually mature males were introduced per group, and therapy continued for seven additional days. Serum malondialdehyde (MDA), total glutathione (tGSH), prolactin, and anti-Mullerian hormone (AMH) levels were measured, and fertility outcomes were evaluated. Results: In single-drug groups, MDA increased and tGSH decreased, but detectable infertility was not recorded. Prolactin remained stable except in the pyrazinamide group, where it declined. Dual-drug regimens increased oxidative imbalance; fertility failure occurred only in pyrazinamide-lacking groups and was accompanied by higher prolactin and lower AMH. Triple and quadruple combinations produced prominent oxidative imbalance. In triple-drug regimens, infertility was lower in pyrazinamide-containing groups than in the pyrazinamide-free group, but this pattern was not maintained in the quadruple regimen. Fertility impairment was not consistently aligned with the degree of oxidative stress and may involve prolactin and AMH alterations. Conclusions: These findings suggest that reproductive impairment under these experimental conditions may involve endocrine alterations and cannot be explained solely by serum oxidative imbalance. Pyrazinamide-associated fertility preservation appeared context-dependent and requires further confirmation in larger mechanistic studies with broader reproductive and endocrine assessment.

## 1. Introduction

Tuberculosis remains a major global public health problem and continues to require prolonged multidrug treatment. According to the World Health Organization, an estimated 10.7 million people developed tuberculosis worldwide in 2024, and approximately 1.23 million deaths were attributed to the disease [1]. Although first-line antituberculosis regimens are essential for disease control and prevention of resistance, their prolonged and combined use raises concerns regarding systemic toxicity and potential effects on reproductive health [2,3]. Rifampicin, isoniazid, pyrazinamide, and ethambutol are anti-tuberculosis agents that have received Food and Drug Administration (FDA) approval for the treatment of *Mycobacterium tuberculosis* infections [2]. Due to the unique characteristics of *Mycobacterium tuberculosis*, tuberculosis therapy should initially begin with a combination of antibiotics. This approach prevents the development of resistant strains [3]. The combination and duration of the drugs used for therapy depend on whether the patient has active or latent disease [4]. The mechanisms of action of these drugs differ from one another. Rifampicin exerts its effect by inhibiting DNA-dependent RNA polymerase [2,5]. Isoniazid is a prodrug that exerts its therapeutic effect after being converted by catalase-peroxidase into an active metabolite, which inhibits the biosynthesis of mycolic acid [6,7]. Pyrazinamide is converted to its active form, pyrazinoic acid, and exerts its effects by inhibiting bacterial trans-translation and possibly coenzyme A synthesis [8]. Ethambutol exerts its antimycobacterial activity by suppressing arabinosyltransferase, an enzyme essential for cell wall biosynthesis in *Mycobacterium tuberculosis* [9]. Rifampicin, isoniazid, pyrazinamide, ethambutol and their combinations (RIPECs) are considered the standard first-line therapy for tuberculosis, owing to their proven efficacy and acceptable safety profile compared to other available regimens [10,11]. However, despite their therapeutic importance, first-line antituberculosis drugs are associated with systemic adverse effects. Hepatotoxicity is among the best-characterized toxicities of rifampicin, isoniazid, pyrazinamide, and ethambutol [12,13], and these systemic toxic effects are frequently linked to oxidative stress and drug-related metabolic burden. Such findings raise the possibility that tissues other than the liver, including reproductive organs, may also be affected during antituberculosis drug exposure. Rifampicin and isoniazid have been reported to exert toxic effects on the reproductive system of female rats [14]. Recent evidence suggests that ethambutol exerts toxic effects on the ovaries, uterus, and placenta through oxidative stress mechanisms, leading to a reduction in pregnancy rate [15]. Although the effects of some first-line antituberculosis drugs on male reproductive parameters have been investigated [16,17], evidence regarding their effects on the female reproductive system remains limited. This gap is particularly important because female reproductive function depends on coordinated ovarian reserve, endocrine regulation, oocyte quality, uterine receptivity, and implantation processes, all of which may be vulnerable to systemic drug toxicity and oxidative stress. Moreover, it remains unclear whether reproductive alterations are caused by individual drugs or by interactions among drugs used in combination therapy. Therefore, the limited and inconsistent data on female reproductive toxicity constitute a central justification for the present study. Experimental evidence from another study suggests that anti-tuberculosis therapy compromises ovarian function by reducing follicular reserve and altering oocyte integrity [18]. A recent study revealed oxidative damage in the uterus and ovaries following antituberculosis combination treatment, supported by both biochemical and histopathological findings [19]. While previous studies suggest that antituberculosis drugs and their combination therapy may negatively impact female reproductive functions, the underlying mechanisms responsible for these effects remain insufficiently understood. Moreover, whether these toxic effects are drug-specific or result from the interaction of agents within the combination therapy remains to be determined. The aim of this study is to investigate the effects of rifampicin, isoniazid, pyrazinamide, ethambutol, and RIPEC on oxidative/antioxidative and endocrine parameters in female rats and to examine the relationship between these parameters and potential reproductive dysfunction.

## 2. Materials and Methods

### 2.1. Animals

A total of 96 female and 32 male albino Wistar rats (aged 9–10 weeks; body weight: 268–277 g) were included in the study. All animals were obtained from the Experimental Animals Application and Research Center of Erzincan Binali Yıldırım University (Erzincan, Turkey). Female rats were randomly allocated into sixteen experimental groups (*n* = 6 per group) to achieve comparable baseline body weights across groups.

During a one-week acclimatization period, animals were housed in standard wire cages (20 cm × 35 cm × 55 cm; floor area: 1925 cm^2^), with six animals per cage. Environmental conditions were strictly controlled, including a 12 h light/dark cycle, an ambient temperature of 22 ± 2 °C, and relative humidity maintained between 30% and 70%. Animals were provided ad libitum access to tap water and standard laboratory chow (Bayramoglu Stock Company, Erzurum, Turkey) throughout the study.

The experimental protocol was conducted in strict accordance with Directive 2010/63/EU of the European Parliament on the protection of animals used for scientific purposes and adhered to the ARRIVE (Animal Research: Reporting of In Vivo Experiments) guidelines [20]. Every effort was made to minimize animal suffering and to reduce the number of animals used to the minimum required to achieve scientific validity.

### 2.2. Reagents and Chemicals

All reagents and chemicals used in this study were of analytical grade and were obtained from certified commercial suppliers. The pharmacological agents included isoniazid (I.N.H.^®^, 300 mg tablet; Catalog No.: 8699828010258), rifampicin (Rifcap^®^, 300 mg capsule; Catalog No.: 8699828150169), pyrazinamide (Pirazinid^®^, 500 mg tablet; Catalog No.: 8699828010326), and ethambutol hydrochloride (Miambutol^®^, 500 mg tablet; Catalog No.: 8699828010302), all of which were supplied by Kocak Farma Pharmaceutical and Chemical Industry Inc. (Istanbul, Turkey).

### 2.3. Experimental Design and Randomization

Sample size was determined in accordance with the principles of the 4R framework (Reduction, Refinement, Replacement, and Responsibility) [21], with the objective of using the minimum number of animals necessary to achieve scientifically valid and reproducible outcomes. Prior to the initiation of experimental procedures, exclusion criteria were prospectively defined and categorized into pre-experimental and peri-/post-experimental phases. Exclusion criteria were categorized as pre-experimental or peri-/post-experimental in order to distinguish baseline health or suitability-related factors from complications or protocol deviations that could occur during treatment, sample collection, or outcome assessment. Pre-experimental exclusion criteria included abnormal body posture, reduced spontaneous activity, or injuries resulting from aggressive interactions among cage mates. Peri-experimental and post-experimental exclusion criteria comprised unexpected mortality prior to the planned study endpoints; complications associated with drug administration; procedural errors, including unsuccessful oral gavage or administration-related failures; deviations from the predefined treatment protocol or incomplete administration of study compounds; body weight loss exceeding 15–20% of baseline values; clinical signs of dehydration or systemic illness; and severe distress indicative of uncontrolled pain or suffering, including persistent vocalization or self-injurious behavior. In addition, loss of sample integrity during collection or processing that could compromise the reliability of downstream analyses was considered grounds for exclusion.

All predefined exclusion criteria were systematically monitored throughout the experimental period and during subsequent data evaluation. No animals met any of the exclusion criteria at any stage of the study; therefore, all animals were included in the final analyses. Animals were allocated to experimental groups using a random number table to ensure unbiased assignment. To further minimize potential confounding factors and systematic sources of bias, each cage and each animal was assigned a unique numerical identification code that was maintained consistently throughout the study period. All outcome assessments were performed by investigators blinded to group allocation.

### 2.4. Experimental Groups

As described above, the 96 female rats were randomly allocated into 16 experimental groups, with six animals in each group. These groups consisted of one healthy control group, four single-drug treatment groups, six dual-drug combination groups, four triple-drug combination groups, and one quadruple-drug combination group. The healthy control group was included once among the 16 experimental groups and served as the common reference group for all comparisons in Test 1, Test 2, and Test 3. It is presented within each test only to facilitate interpretation of the corresponding single-, dual-, and multi-drug comparisons. Animals in the control group only received a vehicle and were maintained under the same experimental conditions as the treatment groups. In the first experimental design (Test 1), the four single-drug treatment groups receiving isoniazid (ISO), rifampicin (RFM), pyrazinamide (PZD), or ethambutol hydrochloride (ETH) were compared with the common healthy control group (CG).

In the second experimental design (Test 2), all possible pairwise combinations among the four first-line antituberculosis drugs were evaluated, comprising ISO + RFM, ISO + PZD, ISO + ETH, RFM + PZD, RFM + ETH, and PZD + ETH. Although these two-drug pairings do not represent complete standard clinical treatment regimens when used alone, they were included to systematically assess whether specific drug–drug interactions contribute differently to oxidative imbalance, endocrine alterations, or fertility outcomes.

In the third experimental design (Test 3), animals were assigned to five groups receiving triple or quadruple drug combinations, namely ISO + RFM + PZD, ISO + RFM + ETH, ISO + PZD + ETH, RFM + PZD + ETH, and the full four-drug regimen (ISO + RFM + PZD + ETH).

### 2.5. Experimental Procedure

#### 2.5.1. Single-Drug Treatments (Test 1)

In Test 1, four groups received monotherapy with isoniazid (ISO; 50 mg/kg), rifampicin (RFM; 50 mg/kg), pyrazinamide (PZD; 250 mg/kg), or ethambutol hydrochloride (ETH; 30 mg/kg), with six animals allocated to each group (*n* = 6). All treatments were administered once daily by oral gavage. The healthy control group (CG; *n* = 6) received an equivalent volume of distilled water via the same route. Treatment was continued for 28 consecutive days. The selected doses and route of administration were based on previously published experimental studies in rats demonstrating that isoniazid [22], rifampicin [22], pyrazinamide [23], and ethambutol [24] can induce reproducible subacute oxidative tissue damage under controlled experimental conditions. These doses were therefore selected to establish an experimental toxicity model suitable for comparative evaluation of oxidative, endocrine, and fertility-related outcomes. They were not intended to directly reproduce clinically equivalent human therapeutic exposures. Therefore, the translational relevance of these findings should be interpreted cautiously, considering interspecies differences in pharmacokinetics, metabolism, tissue distribution, and drug sensitivity. In addition to oxidative toxicity models, previous studies have reported that first-line antituberculosis drugs and their combinations may impair reproductive function, alter endocrine parameters, reduce ovarian reserve and oocyte quality, and induce oxidative injury in ovarian and uterine tissues [13,14,17,18] However, direct dose–response data linking these specific experimental doses to female reproductive toxicity remain limited, particularly for pyrazinamide.

#### 2.5.2. Dual-Drug Treatments (Test 2)

In Test 2, six groups received dual-drug regimens once daily by oral gavage at the same doses used in Test 1: isoniazid + rifampicin (ISO + RFM; *n* = 6), isoniazid + pyrazinamide (ISO + PZD; *n* = 6), isoniazid + ethambutol hydrochloride (ISO + ETH; *n* = 6), rifampicin + pyrazinamide (RFM + PZD; *n* = 6), rifampicin + ethambutol hydrochloride (RFM + ETH; *n* = 6), and pyrazinamide + ethambutol hydrochloride (PZD + ETH; *n* = 6). Treatment was continued for 28 consecutive days.

#### 2.5.3. Triple and Quadruple Drug Combination Treatments (Test 3)

In Test 3, the following triple and quadruple drug combinations were administered once daily by oral gavage using the same doses and procedures described above: isoniazid + rifampicin + pyrazinamide (ISO + RFM + PZD; *n* = 6), isoniazid + rifampicin + ethambutol hydrochloride (ISO + RFM + ETH; *n* = 6), isoniazid + pyrazinamide + ethambutol hydrochloride (ISO + PZD + ETH; *n* = 6), rifampicin + pyrazinamide + ethambutol hydrochloride (RFM + PZD + ETH; *n* = 6), and isoniazid + rifampicin + pyrazinamide + ethambutol hydrochloride (ISO + RFM + PZD + ETH; *n* = 6). Treatment continued for 28 consecutive days.

At the end of the 28-day treatment period, sexually mature male rats were introduced into each group of six females at a ratio of two males per group. Drug administration continued for an additional 7 days during cohabitation. This fixed 7-day cohabitation period was selected because the estrous cycle in rats generally lasts approximately 4–5 days, allowing the mating period to cover at least one complete estrous cycle and a potential fertile phase. At the end of the extended treatment period, blood samples were collected from the lateral tail vein under standard aseptic conditions. Briefly, the tail was gently warmed to facilitate venous dilation, and blood was obtained by tail-vein puncture using appropriate sterile sampling equipment. The collected blood samples were processed for serum separation and subsequently used for the determination of MDA, tGSH, prolactin, and AMH levels. After blood sampling, female rats were separated from the males after the fixed 7-day cohabitation period, regardless of whether mating had been confirmed by vaginal plug or sperm detection. Animals that failed to conceive or deliver within one month after male separation were classified as infertile. Experimental outcomes were evaluated through intergroup comparisons.

### 2.6. Biochemical Analyses

#### 2.6.1. Determination of Serum Oxidant, Antioxidant, and Protein Levels

Malondialdehyde (MDA) and total glutathione (tGSH) levels in the collected blood samples were quantified using commercially available Enzyme-Linked Immunosorbent Assay (ELISA) kits designed for experimental animals (MDA, Catalog No.: 10009055; tGSH, Catalog No.: 703002; Cayman Chemical Co., Ann Arbor, MI, USA), in accordance with the manufacturer’s instructions. Total protein concentrations were determined by the Bradford method [25], which is based on the binding of Coomassie Brilliant Blue G-250 dye to proteins. The dye–protein complex was measured spectrophotometrically at an absorbance of 595 nm.

#### 2.6.2. Determination of Serum Prolactin Levels

Serum prolactin levels were measured using the Abbott Architect immunoassay analyzer (Abbott Medical Inc., Abbott Park, IL, USA) in conjunction with the Architect Prolactin kit (Catalog No: 7K7625, Abbott Medical Inc., Abbott Park, IL, USA).

#### 2.6.3. Determination of Serum AMH Levels

Serum AMH levels in rat samples were measured using AMH assay kits (Unicell-AMH, Yhlo Biotech Co., Ltd., Shenzhen, China) based on an immunochromatographic sandwich method that produces a fluorescent signal proportional to the AMH concentration. According to the manufacturer’s prospectus, the kit has an analytical sensitivity and measurement range of 0.1–25.0 ng/mL.

### 2.7. Statistical Analysis

All statistical analyses were performed using IBM SPSS^®^ Statistics for Windows (Version 27.0; IBM Corp., Armonk, NY, USA, 2020). Graphical representations were generated using GraphPad Prism^®^ (Version 8.0.1; GraphPad Software, San Diego, CA, USA, 2018). Data are presented as mean ± standard error of the mean (SEM). The Shapiro–Wilk test was used to assess normality of data distribution (Tables S1–S3), and Levene’s test was applied to evaluate homogeneity of variances (Tables S4–S6). When the assumption of homogeneity of variances was satisfied, intergroup differences were analyzed using one-way analysis of variance (ANOVA) followed by Tukey’s honestly significant difference (HSD) post hoc test. When this assumption was violated, Welch’s ANOVA followed by the Games–Howell post hoc test was employed. A *p*-value < 0.05 was considered statistically significant.

## 3. Results

### 3.1. Effects of Single-Drug Treatments on Oxidative Stress, Endocrine Markers, and Fertility (Test 1)

**Figure 1:**
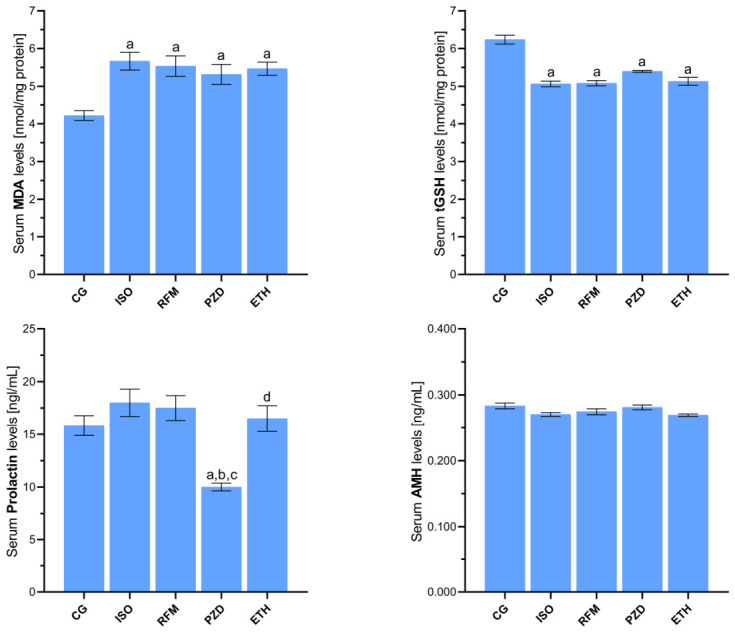
Effects of single drug treatments with isoniazid, rifampicin, pyrazinamide, and ethambutol on rat serum MDA, tGSH, prolactin, and AMH levels assessed in Test 1. Footnotes: Data are presented as mean ± SEM (standard error of the mean). a indicates *p* < 0.05 vs. CG, b indicates *p* < 0.001 vs. ISO, c indicates *p* < 0.001 vs. RFM, and d indicates *p* < 0.05 vs. PZD. All statistical analyses were performed using one-way ANOVA test followed by Tukey’s honestly significant difference (HSD) test. For all groups, *n* = 6. Abbreviations: CG, control group; ISO, isoniazid-only group; RFM, rifampicin-only group; PZD, pyrazinamide-only group; ETH, ethambutol-only group; MDA, malondialdehyde; tGSH, total glutathione; AMH, anti-Mullerian hormone.

In the groups where first-line antituberculosis drugs were administered individually, serum oxidative parameters were increased, whereas antioxidant parameters were decreased relative to the healthy control group. Specifically, MDA levels were significantly higher in all drug-treated groups compared with the control group: CG, 4.22 ± 0.13; ISO, 5.67 ± 0.23; RFM, 5.53 ± 0.27; PZD, 5.32 ± 0.27; and ETH, 5.47 ± 0.18. Post hoc analysis showed statistically significant increases in MDA levels in the ISO, RFM, PZD, and ETH groups compared with the control group, respectively (CG vs. ISO, *p* < 0.001; CG vs. RFM, *p* = 0.003; CG vs. PZD, *p* = 0.014; CG vs. ETH, *p* = 0.004). Conversely, tGSH levels were significantly decreased in all single-drug treatment groups compared with the control group: CG, 6.24 ± 0.12; ISO, 5.06 ± 0.08; RFM, 5.08 ± 0.07; PZD, 5.40 ± 0.03; and ETH, 5.13 ± 0.11; all comparisons versus CG, *p* < 0.001. However, no statistically significant differences were detected among the single-drug treatment groups themselves with respect to MDA or tGSH levels (Figure 1 and Table 1).

Regarding hormonal changes, prolactin concentrations were not significantly altered in the ISO, RFM, or ETH groups compared with the healthy control group (CG, 15.83 ± 0.95; ISO, 18.00 ± 1.29, *p* = 0.604; RFM, 17.50 ± 1.18, *p* = 0.798; ETH, 16.50 ± 1.23, *p* = 0.991). In contrast, prolactin levels were significantly reduced in the PZD group compared with the control group (PZD, 10.00 ± 0.37; *p* = 0.005). Prolactin levels in the PZD group were also significantly lower than those in the ISO, RFM, and ETH groups (*p* < 0.001, *p* < 0.001, and *p* = 0.002, respectively). Anti-Mullerian hormone (AMH) levels remained largely stable across the single-drug treatment groups (CG, 0.283 ± 0.00; ISO, 0.270 ± 0.00; RFM, 0.275 ± 0.00; PZD, 0.281 ± 0.00; ETH, 0.269 ± 0.00). Although the overall ANOVA indicated a significant group effect for AMH, post hoc comparisons did not reveal statistically significant differences between the control group and any single-drug treatment group or among the single-drug groups themselves (Figure 1 and Table 1). No overt fertility failure was recorded in the single-drug treatment groups under the present experimental conditions, as all animals in these groups conceived and/or delivered within the observation period. However, given the relatively small sample size, *n* = 6 per group, and the absence of detailed estrous-cycle monitoring, subtle or subclinical reproductive alterations cannot be excluded (Table 2).

### 3.2. Effects of Dual-Drug Combinations on Oxidative Stress, Endocrine Markers, and Fertility (Test 2)

**Figure 2:**
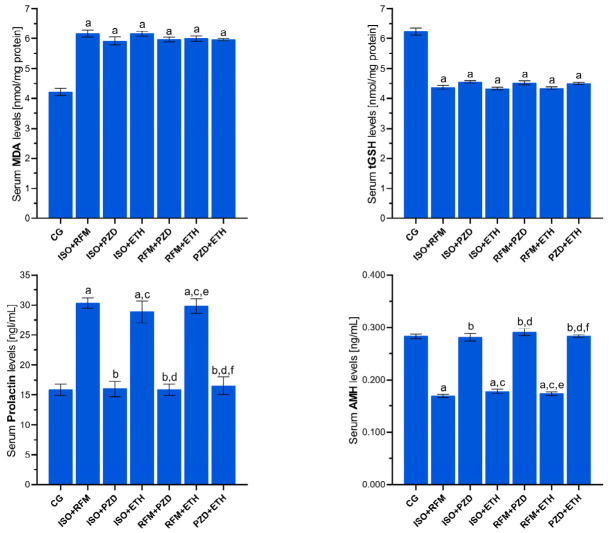
Effects of dual drug regimens with isoniazid, rifampicin, pyrazinamide, and ethambutol on serum MDA, tGSH, prolactin, and AMH levels in rats during Test 2. Footnotes: Data are presented as mean ± SEM (standard error of the mean). a indicates *p* < 0.001 vs. CG, b indicates *p* < 0.001 vs. ISO + RFM, c indicates *p* < 0.001 vs. ISO + PZD, d indicates *p* < 0.001 vs. ISO + ETH, e indicates *p* < 0.001 vs. RFM + PZD, and f indicates *p* < 0.001 vs. RFM + ETH. All statistical analyses were performed using one-way ANOVA test followed by Tukey’s honestly significant difference (HSD) test. For all groups, *n* = 6. Abbreviations: CG, control group; ISO + RFM, isoniazid + rifampicin group; ISO + PZD, isoniazid + pyrazinamide group; ISO + ETH, isoniazid + ethambutol group; RFM + PZD, rifampicin + pyrazinamide group; RFM + ETH, rifampicin + ethambutol group; PZD + ETH, pyrazinamide + ethambutol group; MDA, malondialdehyde; tGSH, total glutathione; AMH, anti-Mullerian hormone.

In Test 2, the primary statistical comparisons were performed among the healthy control group and the dual antituberculosis drug combination groups. Serum MDA levels were significantly increased in all dual-drug combination groups compared with the healthy control group (CG, 4.22 ± 0.13; ISO + RFM, 6.17 ± 0.13; ISO + PZD, 5.92 ± 0.13; ISO + ETH, 6.17 ± 0.08; RFM + PZD, 5.97 ± 0.07; RFM + ETH, 6.00 ± 0.09; PZD + ETH, 5.96 ± 0.04; all comparisons vs. CG, *p* < 0.001). Conversely, serum tGSH levels were significantly decreased in all dual-drug combination groups compared with the control group (CG, 6.24 ± 0.12; ISO + RFM, 4.38 ± 0.06; ISO + PZD, 4.55 ± 0.04; ISO + ETH, 4.33 ± 0.05; RFM + PZD, 4.52 ± 0.07; RFM + ETH, 4.35 ± 0.05; PZD + ETH, 4.50 ± 0.04; all comparisons vs. CG, *p* < 0.001). However, no statistically significant differences were detected among the dual-drug combination groups themselves with respect to MDA or tGSH levels (Figure 2 and Table 3).

When hormonal parameters were examined in Test 2, PZD-containing dual combinations showed prolactin and AMH levels that were not significantly different from those of the healthy control group. Prolactin levels were comparable between the control group and the ISO + PZD, RFM + PZD, and PZD + ETH groups (CG, 15.83 ± 0.95; ISO + PZD, 16.00 ± 1.29, *p* = 1.000; RFM + PZD, 15.83 ± 0.95, *p* = 1.000; PZD + ETH, 16.50 ± 1.48, *p* = 1.000). Similarly, AMH levels did not differ significantly between the control group and the PZD-containing dual-combination groups (CG, 0.283 ± 0.00; ISO + PZD, 0.281 ± 0.01, *p* = 1.000; RFM + PZD, 0.291 ± 0.01, *p* = 0.923; PZD + ETH, 0.283 ± 0.00, *p* = 1.000). In contrast, dual combinations lacking PZD were associated with significantly elevated prolactin levels compared with the control group (ISO + RFM, 30.33 ± 0.88; ISO + ETH, 28.83 ± 1.82; RFM + ETH, 29.83 ± 1.25; all comparisons vs. CG, *p* < 0.001) and significantly reduced AMH levels (ISO + RFM, 0.169 ± 0.00; ISO + ETH, 0.178 ± 0.00; RFM + ETH, 0.174 ± 0.00; all comparisons vs. CG, *p* < 0.001). Although no pooled subgroup analysis was performed to compare all PZD-containing versus all non-PZD-containing combinations, post hoc pairwise comparisons within Test 2 showed that PZD-lacking dual regimens had significantly higher prolactin and lower AMH levels than PZD-containing dual regimens in the relevant comparisons (*p* < 0.001). In contrast, MDA and tGSH levels did not differ significantly among the dual-drug combinations. Within the limits of the present sample size, fertility failure was recorded only in the PZD-lacking dual-combination groups, affecting 33–50% of animals, whereas no fertility failure was recorded in the PZD-containing dual-combination groups (Table 2).

### 3.3. Effects of Triple and Quadruple Drug Combinations on Oxidative Stress, Endocrine Markers, and Fertility (Test 3)

**Figure 3:**
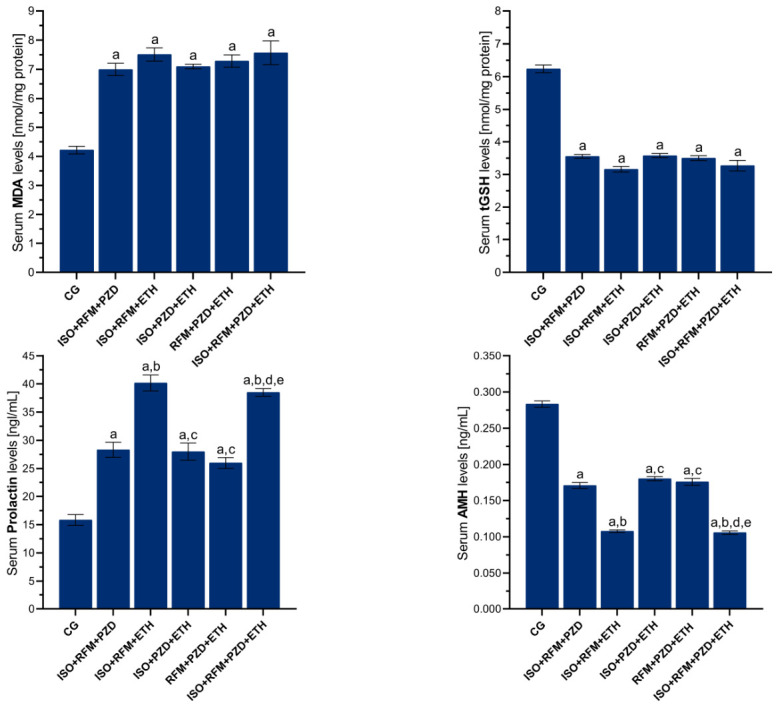
Effects of triple and quadruple combinations of isoniazid, rifampicin, pyrazinamide, and ethambutol on rat serum MDA, tGSH, prolactin, and AMH levels assessed in Test 3. Footnotes: Data are presented as mean ± SEM (standard error of the mean). a indicates *p* < 0.05 vs. CG, b indicates *p* < 0.001 vs. ISO + RFM + PZD, c indicates *p* < 0.001 vs. ISO + RFM + ETH, d indicates *p* < 0.001 vs. ISO + PZD + ETH, e indicates *p* < 0.001 vs. RFM + PZD + ETH. Statistical analyses were conducted using Welch’s ANOVA followed by the Games–Howell test for MDA, while one-way ANOVA followed by Tukey’s honestly significant difference (HSD) test was applied for tGSH, prolactin, and AMH. For all groups, *n* = 6. Abbreviations: CG, control group; ISO + RFM + PZD, isoniazid + rifampicin + pyrazinamide group; ISO + RFM + ETH, isoniazid + rifampicin + ethambutol group; ISO + PZD + ETH, isoniazid + pyrazinamide + ethambutol group; RFM + PZD + ETH, rifampicin + pyrazinamide + ethambutol group; ISO + RFM + PZD + ETH, isoniazid + rifampicin + pyrazinamide + ethambutol group; MDA, malondialdehyde; tGSH, total glutathione; AMH, anti-Mullerian hormone.

In Test 3, the primary statistical comparisons were performed among the shared healthy control group and the triple- and quadruple-drug combination groups. Serum MDA levels were significantly increased in all triple- and quadruple-drug combination groups compared with the control group: CG, 4.22 ± 0.13; ISO + RFM + PZD, 7.00 ± 0.21, *p* < 0.001; ISO + RFM + ETH, 7.51 ± 0.23, *p* < 0.001; ISO + PZD + ETH, 7.10 ± 0.08, *p* < 0.001; RFM + PZD + ETH, 7.29 ± 0.21, *p* < 0.001; and ISO + RFM + PZD + ETH, 7.57 ± 0.41, *p* = 0.002. Similarly, serum tGSH levels were significantly decreased in all triple- and quadruple-drug combination groups compared with the control group: CG, 6.24 ± 0.12; ISO + RFM + PZD, 3.56 ± 0.06; ISO + RFM + ETH, 3.16 ± 0.08; ISO + PZD + ETH, 3.58 ± 0.07; RFM + PZD + ETH, 3.50 ± 0.08; and ISO + RFM + PZD + ETH, 3.27 ± 0.16; all comparisons versus CG, *p* < 0.001. However, no statistically significant differences were detected among the triple- and quadruple-drug combination groups themselves with respect to MDA or tGSH levels (Figure 3 and Table 4).

Evaluation of hormonal parameters showed that prolactin levels were significantly increased and AMH levels were significantly decreased in all triple- and quadruple-drug combination groups compared with the healthy control group (all comparisons vs. CG, *p* < 0.001). Prolactin levels were as follows: CG, 15.83 ± 0.95; ISO + RFM + PZD, 28.33 ± 1.33; ISO + RFM + ETH, 40.17 ± 1.42; ISO + PZD + ETH, 28.00 ± 1.53; RFM + PZD + ETH, 26.00 ± 0.97; and ISO + RFM + PZD + ETH, 38.50 ± 0.67. AMH levels were as follows: CG, 0.283 ± 0.00; ISO + RFM + PZD, 0.171 ± 0.00; ISO + RFM + ETH, 0.108 ± 0.00; ISO + PZD + ETH, 0.180 ± 0.00; RFM + PZD + ETH, 0.176 ± 0.00; and ISO + RFM + PZD + ETH, 0.106 ± 0.00. Among the combination groups, ISO + RFM + ETH and ISO + RFM + PZD + ETH showed significantly higher prolactin levels and significantly lower AMH levels than the PZD-containing triple-drug regimens, namely ISO + RFM + PZD, ISO + PZD + ETH, and RFM + PZD + ETH, in the relevant pairwise comparisons (*p* < 0.001). No statistically significant differences in prolactin or AMH levels were detected between ISO + RFM + ETH and ISO + RFM + PZD + ETH. Within the limits of the present sample size, fertility failure was recorded in 33% of animals in the PZD-containing triple-drug groups, whereas complete fertility failure was recorded in the ISO + RFM + ETH and ISO + RFM + PZD + ETH groups (Table 2).

## 4. Discussion

The mechanisms through which first-line antituberculosis drugs and their combinations may affect female reproductive function have not yet been fully elucidated. It also remains unclear whether these effects are primarily related to the pharmacological properties of individual agents or to drug–drug interactions occurring during combination therapy. In the present pilot study, we investigated the effects of isoniazid, rifampicin, pyrazinamide, and ethambutol, administered either individually or in different combinations, on selected reproductive endpoints in female rats, including fertility outcome, serum oxidant/antioxidant balance, prolactin, and AMH levels. Although first-line antituberculosis drugs are essential components of tuberculosis treatment, previous studies have suggested that these agents may adversely affect reproductive function when administered alone or in combination [26,27,28]. However, evidence regarding their effects on the female reproductive system remains limited and sometimes conflicting [29]. In particular, combination therapy has been reported to intensify reproductive adverse effects compared with single-drug exposure [30,31]. The findings of the present study partially support this claim. No overt fertility failure was recorded in the single-drug treatment groups, despite significant increases in MDA and decreases in tGSH. In contrast, fertility impairment emerged in selected dual-drug combinations and became more frequent in triple and quadruple regimens, indicating that combination therapy may increase reproductive risk compared with single-agent exposure. However, this effect was not uniform across all regimens. Fertility was relatively preserved in some pyrazinamide-containing combinations, whereas more severe fertility impairment was observed in combinations associated with elevated prolactin and reduced AMH levels. Therefore, the present findings suggest that combination therapy may exacerbate reproductive adverse effects in a regimen-dependent manner, and that endocrine disruption and ovarian-reserve-related alterations may contribute alongside oxidative stress.

In the present study, healthy female rats were intentionally used to assess the intrinsic reproductive effects of first-line antituberculosis drugs and their combinations while minimizing the confounding effects of tuberculosis-related inflammation, genital tract involvement, nutritional impairment, oxidative stress, and disease-associated endocrine alterations. Tuberculosis itself, particularly genital tuberculosis, may independently impair reproductive function through chronic inflammation, ovarian reserve depletion, altered AMH secretion, endometrial involvement, impaired implantation, and hypothalamic–pituitary–gonadal axis disruption [28,29]. Therefore, using a tuberculosis disease model at this first-step pilot stage could have obscured whether the observed changes were primarily due to drug exposure, infection-related reproductive damage, or infection–drug interactions. Accordingly, the present findings should be interpreted as drug-associated effects under controlled experimental conditions, not as a complete model of tuberculosis-associated infertility. The discussion of tuberculosis-related infertility was therefore included to contextualize the clinical relevance of reproductive endpoints such as fertility outcome, prolactin, and AMH, rather than to imply that the present model reproduces tuberculosis-associated infertility. In this experimental design, the use of healthy female rats allowed the drug-associated effects on oxidative balance, endocrine markers, and fertility outcomes to be evaluated independently of tuberculosis-related reproductive pathology.

Oxidative stress has been proposed as one of the major mechanisms underlying antituberculosis drug-associated reproductive toxicity. Previous preclinical and clinical studies have shown that antituberculosis agents may decrease antioxidant defense, particularly GSH/tGSH levels, and increase MDA, an end-product of lipid peroxidation [14,32]. Oxidative stress is therefore considered an important contributor to drug-induced reproductive damage [22]. Qiao et al. reported that isoniazid increased reactive oxygen species production in female mice, leading to mitochondrial dysfunction, oocyte deterioration, and infertility [33]. Similarly, isoniazid and rifampicin have been shown to reduce GSH levels and increase MDA levels in association with female reproductive toxicity [14]. Ethambutol has also been reported to induce oxidative stress in female reproductive organs and to impair pregnancy outcomes [15]. In addition, histopathological evidence supports ovarian and uterine injury following first-line antituberculosis drug exposure [19]. However, not all published findings are consistent; for example, one pilot clinical study reported decreased serum MDA and increased GSH levels in patients receiving first-line antituberculosis therapy [34].

Consistent with the oxidative toxicity hypothesis, our results showed that each single-drug treatment significantly increased serum MDA levels and decreased tGSH levels compared with the healthy control group. However, no significant differences were observed among these drug groups in oxidant levels, such as MDA, or antioxidant levels, such as tGSH, and no impairment of reproductive function was detected. In the dual combination groups, oxidant levels showed a greater increase and antioxidant levels a more pronounced decrease compared with the single-drug treatment groups. Although differences in oxidant and antioxidant levels among the dual drug combination groups did not reach statistical significance, varying degrees of reproductive dysfunction (infertility) were detected. Notably, while some degree of infertility occurred in most dual-drug groups, all rats remained fertile in the pyrazinamide-containing combinations. The triple and quadruple drug combination groups exhibited the highest MDA levels and the lowest tGSH levels. Although oxidant and antioxidant levels did not differ significantly between these groups, infertility increased more markedly compared with the dual combinations. Notably, fertility was relatively preserved in pyrazinamide-containing regimens, whereas complete infertility occurred in all rats treated with triple or quadruple combinations lacking pyrazinamide.

To further clarify the effects of these antituberculosis drugs on reproductive functions, in addition to biochemical analyses, prolactin and AMH levels were assessed. Prolactin is an anterior pituitary hormone with significant effects, including the initiation and maintenance of lactation, implantation of pregnancy, and proliferation and differentiation of mammary gland cells [35]. Elevated prolactin levels may suppress hypothalamic gonadotropin-releasing hormone release, thereby impairing follicular maturation, ovulation, and luteal function [36]. Hyperprolactinemia is also recognized as an important endocrine cause of infertility and ovulatory dysfunction [37,38]. In the present study, single-drug administration did not significantly alter prolactin levels, except for pyrazinamide, which reduced prolactin concentrations. The mechanism underlying this reduction remains unclear. Prolactin secretion is primarily controlled by tonic inhibitory dopaminergic signaling from the hypothalamus and may also be influenced by stress-related neuroendocrine responses, hepatic drug metabolism, and alterations in the hypothalamic–pituitary–gonadal axis. Therefore, the lower prolactin level observed in the pyrazinamide group may reflect an indirect neuroendocrine or metabolic effect rather than a direct action on pituitary lactotroph cells. In addition, the relatively preserved prolactin profile observed in pyrazinamide-containing dual combinations may suggest that pyrazinamide modifies the endocrine response to certain drug combinations. However, because dopamine, GnRH, LH, FSH, estradiol, progesterone, and pituitary histology were not evaluated in the present study, this interpretation remains hypothetical. In contrast, dual combinations lacking pyrazinamide markedly increased prolactin levels, whereas pyrazinamide-containing dual combinations showed prolactin values closer to those of the control group. A similar pattern was observed in triple regimens, in which prolactin elevation was more pronounced in the pyrazinamide-free combination. These findings are biologically plausible, as previous reports have suggested that rifampicin and isoniazid may interfere with hormone metabolism or prolactin regulation [39,40]. Our findings are partly consistent with previous studies reporting reproductive toxicity, oxidative stress, endocrine disruption, and impaired fertility outcomes following exposure to isoniazid, rifampicin, ethambutol, or fixed-dose antituberculosis combinations [14,15,18,19,30,31,33]. Ezeuko and Ataman reported antifertility effects of isoniazid and rifampicin in adult female rats [14], while Adebayo et al. demonstrated that first-line antituberculosis drugs disrupted endocrine balance and induced ovarian and uterine oxidative stress [19]. Similarly, Awodele et al. reported that combined fixed-dose antituberculosis drugs altered reproductive functions with oxidative stress involvement [30], and Rao et al. showed that antituberculosis therapy reduced ovarian reserve and oocyte quality in mice [18]. However, the absence of infertility in the single-drug groups and the partial preservation of fertility in some pyrazinamide-containing combinations observed in our study differ from certain previous reports, possibly due to differences in dose, treatment duration, animal strain, endpoint selection, and experimental design.

To specifically assess the potential toxicity of these antituberculosis drugs on ovarian function, AMH levels in the serum of the animals were measured in this study. AMH is a glycoprotein secreted by granulosa cells of small follicles and is used as a reliable clinical marker of ovarian reserve [41]. Previous studies have shown that low levels of AMH correlate with reduced pregnancy rates [42,43]. Studies in the literature have suggested that genital involvement in latent tuberculosis can lead to a decrease in AMH secretion and ovarian reserve, resulting in infertility [44,45]. Furthermore, some studies have demonstrated that antituberculosis treatment increases AMH levels in women with genital tuberculosis, improving ovarian function and ovarian blood flow [28]. When the findings of our study are examined, the use of antituberculosis drugs alone did not significantly affect AMH levels. However, in the dual combination groups containing pyrazinamide, no significant changes in AMH levels were observed, while a significant decrease was seen in other dual combination groups. AMH levels were markedly reduced in the triple and quadruple drug combination groups relative to the healthy controls. Notably, the inclusion of pyrazinamide in triple-drug regimens was associated with significantly higher AMH levels compared to pyrazinamide-free combinations. The observed reduction in AMH levels has been associated with impaired ovarian reserve [41,42,43]. Moreover, our findings, showing a decrease in AMH levels corresponding to the increase in prolactin levels, are consistent with the data reported in the literature [46].

Fertility outcomes provided an integrated functional endpoint for evaluating reproductive impairment. Infertility was not detected in the healthy control group or in any of the single-drug groups, despite significant oxidative imbalance. In dual combinations, infertility occurred in the isoniazid plus ethambutol, rifampicin plus ethambutol, and isoniazid plus rifampicin groups, whereas all animals remained fertile in pyrazinamide-containing dual combinations. In triple combinations, infertility reached 100% in the pyrazinamide-free regimen, whereas pyrazinamide-containing triple combinations showed a lower infertility rate. However, the quadruple combination did not prevent infertility, indicating that the possible fertility-preserving association observed with pyrazinamide in some combinations may be context-dependent and insufficient when all four drugs are administered together.

Our experimental findings suggest that oxidative stress alone may not fully account for the infertility observed under these experimental conditions. Several additional mechanisms may contribute to impaired reproductive outcomes. Elevated prolactin may suppress hypothalamic gonadotropin-releasing hormone release, thereby impairing LH and FSH secretion, follicular maturation, ovulation, and luteal function [35,36,37,38]. Reduced AMH levels may indicate impaired ovarian reserve and altered granulosa-cell function, both of which are associated with lower reproductive potential [41,42,43]. In addition, antituberculosis drugs may affect oocyte quality, mitochondrial function, follicular development, uterine receptivity, and implantation processes [18,19,31,33]. Estrous cycle disruption, altered estradiol and progesterone levels, changes in gonadotropins, and drug–drug interactions affecting metabolism and tissue exposure may also contribute to the observed infertility. Because these parameters were not comprehensively evaluated in the present study, infertility observed in some combination groups should be interpreted as a multifactorial outcome rather than as a consequence of oxidative stress alone.

The available literature indicates that first-line antituberculosis drugs may impair reproductive function through oxidative stress-related mechanisms when administered alone or in combination [14,15,16,17,19,26,30,31,33]. Histopathological studies have also shown oxidative damage in ovarian and uterine tissues following antituberculosis combination therapy [19]. Ethambutol has been associated with reduced pregnancy rates through oxidative injury in female reproductive organs [15], while rifampicin and isoniazid have been reported to exert toxic effects on female reproductive tissues [14]. However, data regarding the effects of pyrazinamide on female reproductive function remain scarce and inconclusive [16,17]. No formal correlation analysis was performed between oxidative stress parameters and fertility outcomes. Therefore, the observation that fertility impairment was not consistently aligned with MDA elevation or tGSH depletion should be interpreted as a hypothesis rather than as a statistically confirmed relationship. Future studies with larger sample sizes and formal correlation or regression analyses are needed to clarify whether oxidative stress independently predicts fertility outcomes.

It should also be noted that healthy untreated male rats were used for mating in the present design. This approach was selected to minimize the potential confounding effect of male-factor infertility and to evaluate fertility outcomes primarily in relation to drug-exposed females. However, previous studies have shown that first-line antituberculosis drugs and their combinations may impair male reproductive parameters, including spermatogenesis, sperm morphology, and reproductive capacity [16,17,26,30]. Therefore, the present study does not address the potential contribution of paternal drug exposure to fertility outcomes. Future studies including both drug-exposed females and males, together with estrous cycle monitoring, broader hormonal profiling, ovarian and uterine histopathology, and longer post-treatment follow-up, are needed to clarify the mechanisms and reversibility of antituberculosis drug combination-associated reproductive impairment.

Overall, our findings suggest that first-line antituberculosis drug combinations may adversely affect selected reproductive endpoints in female rats, particularly when associated with prolactin elevation and AMH reduction. Although oxidative stress appears to be involved, it does not fully explain the infertility observed under these experimental conditions. These results should therefore be interpreted cautiously as preliminary evidence from a controlled pilot model and should be validated in more comprehensive experimental designs, including tuberculosis disease models and extended reproductive assessments.

## 5. Conclusions

In this pilot study, the single administration of isoniazid, rifampicin, pyrazinamide, or ethambutol induced serum oxidative imbalance in healthy female rats but did not result in detectable infertility under the present experimental conditions. In contrast, selected dual, triple, and quadruple drug combinations were associated with impaired fertility outcomes, particularly in groups showing elevated prolactin and reduced AMH levels. Although oxidative stress appeared to be involved, the degree of serum oxidative imbalance did not fully parallel the observed fertility outcomes, suggesting that endocrine alterations and ovarian-reserve-related mechanisms may also contribute to reproductive impairment under these experimental conditions.

The apparent partial preservation of fertility in some pyrazinamide-containing combinations should be interpreted cautiously, as this effect was not observed in the quadruple regimen and requires confirmation in further studies. Given the pilot nature of the study, the use of healthy animals, and the absence of tuberculosis disease modeling, estrous cycle monitoring, broader hormonal profiling, and histopathological confirmation, these findings should not be directly extrapolated to clinical practice. Further experimental and translational studies are needed to clarify the mechanisms, reversibility, and clinical relevance of antituberculosis drug combination-associated reproductive effects.

## 6. Limitations and Future Perspectives

Despite its strengths, the present study has several limitations that should be considered when interpreting the findings. Healthy female rats were used, and an active or genital tuberculosis model was not established. This approach was deliberately selected to isolate the intrinsic reproductive effects of first-line antituberculosis drugs and their combinations from the confounding effects of tuberculosis-related systemic inflammation, genital tract involvement, nutritional impairment, oxidative stress, and disease-associated endocrine alterations. However, this design does not fully reproduce the clinical setting of tuberculosis, in which reproductive dysfunction may result from the combined effects of infection-related mechanisms and drug exposure. Therefore, future studies using appropriate tuberculosis disease models are needed to clarify potential infection–drug interactions affecting female reproductive function. In addition, healthy untreated male rats were used for mating to minimize the potential confounding influence of male-factor infertility and to allow fertility outcomes to be interpreted primarily in relation to drug-exposed females. Nevertheless, this approach does not reflect clinical conditions in which male partners may also be affected by tuberculosis or exposed to antituberculosis therapy. Since previous studies have reported that first-line antituberculosis drugs may impair male reproductive parameters, future investigations should include experimental designs evaluating both maternal and paternal drug exposure. Another limitation is that the present study did not evaluate whether antioxidant interventions could mitigate drug-associated reproductive alterations. Considering the observed changes in MDA and tGSH levels, future studies investigating antioxidant or cytoprotective strategies may help clarify the contribution of oxidative stress to reproductive impairment. Furthermore, ovarian and uterine tissue analyses were not supported by histopathological confirmation. Although serum oxidative/antioxidative and endocrine markers provided important information, histological evaluation of ovarian follicles, corpus luteum structure, uterine morphology, and implantation-related changes would have strengthened the mechanistic interpretation of the findings. Moreover, daily vaginal cytology was not performed to systematically monitor estrous cycle regularity during the experimental period. Although the animals were sexually mature and maintained under controlled laboratory conditions in which albino Wistar rats typically exhibit regular 4–5-day estrous cycles, subtle variations in cycle length, phase progression, or ovulatory timing cannot be excluded. Therefore, future studies should include detailed estrous cycle monitoring to better define the relationship between drug exposure and ovulatory function. In addition, serum progesterone and estradiol levels, along with gonadotropins such as LH and FSH, were not assessed. Although infertility was associated with altered prolactin and AMH concentrations, broader hormonal profiling would have provided a more comprehensive evaluation of the hypothalamic–pituitary–gonadal axis. Future investigations incorporating gonadotropins and steroid hormones are needed to clarify the full spectrum of endocrine mechanisms underlying treatment-associated infertility. Finally, extended post-treatment follow-up was not performed to determine the long-term persistence or reversibility of the observed infertility. Males were introduced on day 28 of drug administration and co-housed with females for 7 consecutive days while treatment continued. Following male separation, females were monitored for an additional 30 days to assess pregnancy outcomes. Although this design allowed evaluation of reproductive capacity during sustained drug exposure and early follow-up, fertility was not assessed beyond this period. Therefore, longer-term studies are required to determine whether the observed reproductive alterations are reversible after treatment discontinuation.

## Figures and Tables

**Table 1:** Comparison of *p*-values for the effects of individual antituberculosis drugs on oxidant, antioxidant, and hormone levels in rat serum (Test 1).

	Post hoc Test *p*-Values			
Group Comparisons	MDA	tGSH	Prolactin	AMH
CG vs. ISO	<0.001	<0.001	0.604	0.108
CG vs. RFM	0.003	<0.001	0.798	0.428
CG vs. PZD	0.014	<0.001	0.005	0.993
CG vs. ETH	0.004	<0.001	0.991	0.073
ISO vs. RFM	0.993	1.000	0.997	0.921
ISO vs. PZD	0.797	0.071	<0.001	0.237
ISO vs. ETH	0.968	0.978	0.852	1.000
RFM vs. PZD	0.957	0.097	<0.001	0.685
RFM vs. ETH	1.000	0.993	0.961	0.844
PZD vs. ETH	0.989	0.211	0.002	0.168
F value	6.959	34.085	9.292	3.091
df (df1/df2)	4/25	4/25	4/25	4/25
*p*-values	<0.001	<0.001	<0.001	0.034

Footnotes: All statistical analyses were conducted with one-way ANOVA, followed by Tukey’s honestly significant difference (HSD) test for post hoc multiple comparisons. For all groups *n* = 6. Abbreviations: CG, control group; ISO, isoniazid-only group; RFM, rifampicin-only group; PZD, pyrazinamide-only group; ETH, ethambutol-only group; MDA, malondialdehyde; tGSH, total glutathione; AMH, anti-Mullerian hormone; df, degrees of freedom; df1, numerator degrees of freedom; df2, denominator degrees of freedom.

**Table 2:** Fertility outcomes of the experimental groups.

	Experimental Groups	Rats per Group	Number of Fertile Rats	%	Number of Infertile Rats	%
Test 1	CG	6	6	100	0	0
	ISO	6	6	100	0	0
	RFM	6	6	100	0	0
	PZD	6	6	100	0	0
	ETH	6	6	100	0	0
Test 2	CG	6	6	100	0	0
	ISO + RFM	6	3	50	3	50
	ISO + PZD	6	6	100	0	0
	ISO + ETH	6	4	67	2	33
	RFM + PZD	6	6	100	0	0
	RFM + ETH	6	4	67	2	33
	PZD + ETH	6	6	100	0	0
Test 3	CG	6	6	100	0	0
	ISO + RFM + PZD	6	4	67	2	33
	ISO + RFM + ETH	6	0	0	6	100
	ISO + PZD + ETH	6	4	67	2	33
	RFM + PZD + ETH	6	4	67	2	33
	ISO + RFM + PZD + ETH	6	0	0	6	100

Footnotes: For all groups *n* = 6. Abbreviations: CG, control group; ISO, isoniazid; RFM, rifampicin; PZD, pyrazinamide; ETH, ethambutol.

**Table 3:** Comparison of *p*-values for the effects of dual combinations of antituberculosis drugs on oxidant, antioxidant, and hormone levels in rat serum (Test 2).

	Post hoc Test *p*-Values			
Group Comparisons	MDA	tGSH	Prolactin	AMH
CG vs. ISO + RFM	<0.001	<0.001	<0.001	<0.001
CG vs. ISO + PZD	<0.001	<0.001	1.000	1.000
CG vs. ISO + ETH	<0.001	<0.001	<0.001	<0.001
CG vs. RFM + PZD	<0.001	<0.001	1.000	0.923
CG vs. RFM + ETH	<0.001	<0.001	<0.001	<0.001
CG vs. PZD + ETH	<0.001	<0.001	1.000	1.000
ISO + RFM vs. ISO + PZD	0.585	0.486	<0.001	<0.001
ISO + RFM vs. ISO + ETH	1.000	0.999	0.979	0.869
ISO + RFM vs. RFM + PZD	0.800	0.691	<0.001	<0.001
ISO + RFM vs. RFM + ETH	0.903	1.000	1.000	0.996
ISO + RFM vs. PZD + ETH	0.782	0.804	<0.001	<0.001
ISO + PZD vs. ISO + ETH	0.585	0.244	<0.001	<0.001
ISO + PZD vs. RFM + PZD	1.000	1.000	1.000	0.799
ISO + PZD vs. RFM + ETH	0.997	0.312	<0.001	<0.001
ISO + PZD vs. PZD + ETH	1.000	0.998	1.000	1.000
ISO + ETH vs. RFM + PZD	0.800	0.410	<0.001	<0.001
ISO + ETH vs. RFM + ETH	0.903	1.000	0.998	0.995
ISO + ETH vs. PZD + ETH	0.782	0.531	<0.001	<0.001
RFM + PZD vs. RFM + ETH	1.000	0.497	<0.001	<0.001
RFM + PZD vs. PZD + ETH	1.000	1.000	1.000	0.923
RFM + ETH vs. PZD + ETH	1.000	0.623	<0.001	<0.001
F value	46.690	110.095	33.089	153.533
df (df1/df2)	6/35	6/35	6/35	6/35
*p*-values	<0.001	<0.001	<0.001	<0.001

Footnotes: All statistical analyses were conducted with one-way ANOVA, followed by Tukey’s honestly significant difference (HSD) test for post hoc multiple comparisons. For all groups *n* = 6. Abbreviations: CG, control group; ISO + RFM, isoniazid + rifampicin group; ISO + PZD, isoniazid + pyrazinamide group; ISO + ETH, isoniazid + ethambutol group; RFM + PZD, rifampicin + pyrazinamide group; RFM + ETH, rifampicin + ethambutol group; PZD + ETH, pyrazinamide + ethambutol group; MDA, malondialdehyde; tGSH, total glutathione; AMH, anti-Mullerian hormone; df, degrees of freedom; df1, numerator degrees of freedom; df2, denominator degrees of freedom.

**Table 4:** Comparison of *p*-values for the effects of triple and quadruple combinations of antituberculosis drugs on oxidant, antioxidant, and hormone levels in rat serum (Test 3).

	Post hoc Test *p*-Values			
Group Comparisons	MDA *	tGSH **	Prolactin **	AMH **
CG vs. ISO + RFM + PZD	<0.001	<0.001	<0.001	<0.001
CG vs. ISO + RFM + ETH	<0.001	<0.001	<0.001	<0.001
CG vs. ISO + PZD + ETH	<0.001	<0.001	<0.001	<0.001
CG vs. RFM + PZD + ETH	<0.001	<0.001	<0.001	<0.001
CG vs. ISO + RFM + PZD + ETH	0.002	<0.001	<0.001	<0.001
ISO + RFM + PZD vs. ISO + RFM + ETH	0.588	0.086	<0.001	<0.001
ISO + RFM + PZD vs. ISO + PZD + ETH	0.997	1.000	1.000	0.451
ISO + RFM + PZD vs. RFM + PZD + ETH	0.920	0.999	0.731	0.917
ISO + RFM + PZD vs. ISO + RFM + PZD + ETH	0.803	0.358	<0.001	<0.001
ISO + RFM + ETH vs. ISO + PZD + ETH	0.569	0.061	<0.001	<0.001
ISO + RFM + ETH vs. RFM + PZD + ETH	0.974	0.182	<0.001	<0.001
ISO + RFM + ETH vs. ISO + RFM + PZD + ETH	1.000	0.968	0.916	0.999
ISO + PZD + ETH vs. RFM + PZD + ETH	0.952	0.994	0.836	0.953
ISO + PZD + ETH vs. ISO + RFM + PZD + ETH	0.849	0.281	<0.001	<0.001
RFM + PZD + ETH vs. ISO + RFM + PZD + ETH	0.986	0.583	<0.001	<0.001
F value	68.406 ^a^	137.177	56.661	330.048
df (df1/df2)	5/13.366 ^b^	5/30	5/30	5/30
*p*-values	<0.001	<0.001	<0.001	<0.001

Footnotes: * Statistical analyses were performed using Welch’s ANOVA, followed by the Games–Howell test for post hoc multiple comparisons of MDA. ** Statistical analyses were performed using one-way ANOVA, followed by Tukey’s honestly significant difference (HSD) test for post hoc multiple comparisons of tGSH, prolactin, and AMH. ^a^ indicates asymptotically F distributed. ^b^ indicates Welch ANOVA *p*-values. For all groups, *n* = 6. Abbreviations: CG, control group; ISO + RFM + PZD, isoniazid + rifampicin + pyrazinamide group; ISO + RFM + ETH, isoniazid + rifampicin + ethambutol group; ISO + PZD + ETH, isoniazid + pyrazinamide + ethambutol group; RFM + PZD + ETH, rifampicin + pyrazinamide + ethambutol group; ISO + RFM + PZD + ETH, isoniazid + rifampicin + pyrazinamide + ethambutol group; MDA, malondialdehyde; tGSH, total glutathione; AMH, anti-Mullerian hormone; df, degrees of freedom; df1, numerator degrees of freedom; df2, denominator degrees of freedom.

## Data Availability

The original contributions presented in this study are included in the article/Supplementary Materials. Further inquiries can be directed to the corresponding author.

## Supplementary Materials

The following supporting information can be downloaded at: https://www.mdpi.com/article/10.3390/life16060878/s1, Table S1. Shapiro–Wilk test results evaluating the assumption of normality for biochemical variables measured in rat serum in Test 1; Table S2. Shapiro–Wilk test results evaluating the assumption of normality for biochemical variables measured in rat serum in Test 2; Table S3. Shapiro–Wilk test results evaluating the assumption of normality for biochemical variables measured in rat serum in Test 3; Table S4. Assessment of homogeneity of variances for MDA, tGSH, prolactin, and AMH levels in Test 1; Table S5. Assessment of homogeneity of variances for MDA, tGSH, prolactin, and AMH levels in Test 2; Table S6. Assessment of homogeneity of variances for MDA, tGSH, prolactin, and AMH levels in Test 3.
