# Supplementary material for: Pilot Study on the Effects of First-Line Antituberculosis Drugs and Their Combinations on Selected Reproductive Endpoints in Female Rats
Source: Life (Basel). 2026 May 24;16(6):878. doi: 10.3390/life16060878 (PMC13302617; doi:10.3390/life16060878)
Supplement: Supplementary file 1 [file life-16-00878-s001.zip › Table S1.pdf]

Table S1. Shapiro–Wilk test results evaluating the assumption of normality for biochemical variables measured in rat serum in Test 1

		Shapiro-Wilk	Biochemical Variables			
			**MDA**	**tGSH**	**Prolactin**	**AMH**
Groups	CG	Statistic	0.988	0.946	0.958	0.982
		df	6	6	6	6
		Sig.	0.983	0.712	0.801	0.959
	ISO	Statistic	0.976	0.814	0.979	0.992
		df	6	6	6	6
		Sig.	0.930	0.079	0.949	0.994
	RFM	Statistic	0.940	0.977	0.917	0.917
		df	6	6	6	6
		Sig.	0.658	0.934	0.484	0.484
	PZD	Statistic	0.891	0.861	0.853	0.970
		df	6	6	6	6
		Sig.	0.325	0.192	0.167	0.892
	ETH	Statistic	0.945	0.862	0.992	0.957
		df	6	6	6	6
		Sig.	0.698	0.198	0.993	0.798

Footnotes: The distributions of MDA, tGSH, prolactin, and AMH levels were consistent with the assumptions of normality; hence, group comparisons were conducted using one-way ANOVA.

Abbreviations: CG, control group; ISO, isoniazid-only group; RFM, rifampicin-only group; PZD, pyrazinamide-only group; ETH, ethambutol-only group; MDA, malondialdehyde; tGSH, total glutathione; AMH, anti-Mullerian hormone; df, degrees of freedom; Sig, significance.
