# Supplementary material for: Pilot Study on the Effects of First-Line Antituberculosis Drugs and Their Combinations on Selected Reproductive Endpoints in Female Rats
Source: Life (Basel). 2026 May 24;16(6):878. doi: 10.3390/life16060878 (PMC13302617; doi:10.3390/life16060878)
Supplement: Supplementary file 1 [file life-16-00878-s001.zip › Table S2.pdf]

Table S2. Shapiro–Wilk test results evaluating the assumption of normality for biochemical variables measured in rat serum in Test 2

		Shapiro-Wilk	Biochemical Variables			
			MDA	tGSH	Prolactin	AMH
Groups	CG	Statistic	0.988	0.946	0.958	0.982
		df	6	6	6	6
		Sig.	0.983	0.712	0.801	0.959
	ISO+RFM	Statistic	0.941	0.954	0.983	0.957
		df	6	6	6	6
		Sig.	0.664	0.770	0.964	0.796
	ISO+PZD	Statistic	0.891	0.978	0.979	0.917
		df	6	6	6	6
		Sig.	0.324	0.939	0.949	0.483
	ISO+ETO	Statistic	0.915	0.961	0.912	0.957
		df	6	6	6	6
		Sig.	0.473	0.831	0.451	0.796
	RFM+PZD	Statistic	0.974	0.987	0.958	0.968
		df	6	6	6	6
		Sig.	0.918	0.980	0.801	0.878
	RFM+ETO	Statistic	0.955	0.982	0.963	0.925
		df	6	6	6	6
		Sig.	0.783	0.961	0.846	0.545
	PZD+ETO	Statistic	0.932	0.986	0.979	0.880
		df	6	6	6	6
		Sig.	0.593	0.978	0.949	0.267

Footnotes: The distributions of MDA, tGSH, prolactin, and AMH levels were consistent with the assumptions of normality; hence, group comparisons were conducted using one-way ANOVA.

Abbreviations: CG, control group; ISO+RFM, isoniazid + rifampicin group; ISO+PZD, isoniazid + pyrazinamide group; ISO+ETH, isoniazid + ethambutol group; RFM+PZD, rifampicin + pyrazinamide group; RFM+ETH, rifampicin + ethambutol group; PZD+ETH, pyrazinamide + ethambutol group; MDA, malondialdehyde; tGSH, total glutathione; AMH, anti-Mullerian hormone; df, degrees of freedom; Sig, significance.
