# Supplementary material for: Pilot Study on the Effects of First-Line Antituberculosis Drugs and Their Combinations on Selected Reproductive Endpoints in Female Rats
Source: Life (Basel). 2026 May 24;16(6):878. doi: 10.3390/life16060878 (PMC13302617; doi:10.3390/life16060878)
Supplement: Supplementary file 1 [file life-16-00878-s001.zip › Table S3.pdf]

Table S3. Shapiro–Wilk test results evaluating the assumption of normality for biochemical variables measured in rat serum in Test 3

			Biochemical Variables			
		**Shapiro-Wilk**	**MDA**	**tGSH**	**Prolactin**	**AMH**
Groups	CG	Statistic	0.988	0.946	0.958	0.982
		df	6	6	6	6
		Sig.	0.983	0.712	0.801	0.959
	ISO+RFM+PZD	Statistic	0.915	0.942	0.918	0.910
		df	6	6	6	6
		Sig.	0.472	0.674	0.492	0.439
	ISO+RFM+ETH	Statistic	0.945	0.962	0.961	0.991
		df	6	6	6	6
		Sig.	0.697	0.838	0.830	0.990
	ISO+PZD+ETH	Statistic	0.944	0.988	0.980	0.979
		df	6	6	6	6
		Sig.	0.694	0.984	0.953	0.945
	RFM+PZD+ETH	Statistic	0.955	0.924	0.950	0.935
		df	6	6	6	6
		Sig.	0.781	0.536	0.739	0.620
	ISO+RFM+PZD+ETH	Statistic	0.928	0.880	0.951	0.867
		df	6	6	6	6
		Sig.	0.563	0.267	0.752	0.216

Footnotes: The distributions of MDA, tGSH, prolactin, and AMH levels were consistent with the assumptions of normality; hence, group comparisons were conducted using ANOVA.

Abbreviations: CG, control group; ISO+RFM+PZD, isoniazid + rifampicin + pyrazinamide group; ISO+RFM+ETH, isoniazid + rifampicin + ethambutol group; ISO+PZD+ETH, isoniazid + pyrazinamide + ethambutol group; RFM+PZD+ETH, rifampicin + pyrazinamide + ethambutol group; ISO+RFM+PZD+ETH, isoniazid + rifampicin + pyrazinamide + ethambutol group; MDA, malondialdehyde; tGSH, total glutathione; AMH, anti-Mullerian hormone; df, degrees of freedom; Sig, significance.
