# Supplementary material for: Pilot Study on the Effects of First-Line Antituberculosis Drugs and Their Combinations on Selected Reproductive Endpoints in Female Rats
Source: Life (Basel). 2026 May 24;16(6):878. doi: 10.3390/life16060878 (PMC13302617; doi:10.3390/life16060878)
Supplement: Supplementary file 1 [file life-16-00878-s001.zip › Table S5.pdf]

Table S5. Assessment of homogeneity of variances for MDA, tGSH, prolactin, and AMH levels in Test 2

	Biochemical Variables			
	**MDA**	**tGSH**	**Prolactin**	**AMH**
Levene's statistic	2.008	1.641	0.986	1.512
df1	6	6	6	6
df2	35	35	35	35
Sig.	0.091	0.165	0.449	0.203

Footnotes: Since the assumption of homogeneity of variances was confirmed, Tukey's honestly significant difference (HSD) test was used for the post hoc evaluation of MDA, tGSH, prolactin, and AMH levels in Test 2.

Abbreviations: MDA, malondialdehyde; tGSH, total glutathione; AMH, anti-Mullerian hormone; df1, numerator degrees of freedom; df2, denominator degrees of freedom; Sig, significance.
