# Supplementary material for: Pilot Study on the Effects of First-Line Antituberculosis Drugs and Their Combinations on Selected Reproductive Endpoints in Female Rats
Source: Life (Basel). 2026 May 24;16(6):878. doi: 10.3390/life16060878 (PMC13302617; doi:10.3390/life16060878)
Supplement: Supplementary file 1 [file life-16-00878-s001.zip › Table S6.pdf]

Table S6. Assessment of homogeneity of variances for MDA, tGSH, prolactin, and AMH levels in Test 3

	Biochemical Variables			
	**MDA**	**tGSH**	**Prolactin**	**AMH**
Levene's statistic	2.778	2.081	0.936	1.873
df1	5	5	5	5
df2	30	30	30	30
Sig.	0.035	0.095	0.472	0.129

Footnotes: Since the assumption of homogeneity of variances was not confirmed, Games-Howell test was used for the post hoc evaluation of MDA levels in Test 3. In contrast, as the assumption of homogeneity of variances was confirmed, Tukey's honestly significant difference (HSD) test was also employed for the post hoc evaluation of tGSH, prolactin, and AMH levels in Test 3.

Abbreviations: MDA, malondialdehyde; tGSH, total glutathione; AMH, anti-Mullerian hormone; df1, numerator degrees of freedom; df2, denominator degrees of freedom; Sig, significance.
